# Genetic Variation in Choline-Metabolizing Enzymes Alters Choline Metabolism in Young Women Consuming Choline Intakes Meeting Current Recommendations

**DOI:** 10.3390/ijms18020252

**Published:** 2017-01-26

**Authors:** Ariel B. Ganz, Vanessa V. Cohen, Camille C. Swersky, Julie Stover, Gerardo A. Vitiello, Jessica Lovesky, Jasmine C. Chuang, Kelsey Shields, Vladislav G. Fomin, Yusnier S. Lopez, Sanjay Mohan, Anita Ganti, Bradley Carrier, Olga V. Malysheva, Marie A. Caudill

**Affiliations:** Division of Nutritional Sciences, Cornell University, Ithaca, NY 14853, USA; abg224@cornell.edu (A.B.G.); vcohen20@gmail.com (V.V.C.); ccs229@cornell.edu (C.C.S.); jas683@cornell.edu (J.S.); gav8@cornell.edu (G.A.V.); lovesky.jessica@gmail.com (J.L.); chuangjazz@gmail.com (J.C.C.); shields.kelsey@gmail.com (K.S.); shortforv@yahoo.com (V.G.F.); sanjay.mohan90@gmail.com (S.M.); anitaganti16@gmail.com (A.G.); bradjc23@gmail.com (B.C.); ovm4@cornell.edu (O.V.M.)

**Keywords:** choline, nutritional genomics, nutrigenetics, single nucleotide polymorphisms, pregnancy, lactation, one-carbon metabolism, micronutrient metabolism

## Abstract

Single nucleotide polymorphisms (SNPs) in choline metabolizing genes are associated with disease risk and greater susceptibility to organ dysfunction under conditions of dietary choline restriction. However, the underlying metabolic signatures of these variants are not well characterized and it is unknown whether genotypic differences persist at recommended choline intakes. Thus, we sought to determine if common genetic risk factors alter choline dynamics in pregnant, lactating, and non-pregnant women consuming choline intakes meeting and exceeding current recommendations. Women (*n* = 75) consumed 480 or 930 mg choline/day (22% as a metabolic tracer, choline-d9) for 10–12 weeks in a controlled feeding study. Genotyping was performed for eight variant SNPs and genetic differences in metabolic flux and partitioning of plasma choline metabolites were evaluated using stable isotope methodology. *CHKA* rs10791957, *CHDH* rs9001, *CHDH* rs12676, *PEMT* rs4646343, *PEMT* rs7946, *FMO3* rs2266782, *SLC44A1* rs7873937, and *SLC44A1* rs3199966 altered the use of choline as a methyl donor; *CHDH* rs9001 and *BHMT* rs3733890 altered the partitioning of dietary choline between betaine and phosphatidylcholine synthesis via the cytidine diphosphate (CDP)-choline pathway; and *CHKA* rs10791957, *CHDH* rs12676, *PEMT* rs4646343, *PEMT* rs7946 and *SLC44A1* rs7873937 altered the distribution of dietary choline between the CDP-choline and phosphatidylethanolamine *N*-methyltransferase (PEMT) denovo pathway. Such metabolic differences may contribute to disease pathogenesis and prognosis over the long-term.

## 1. Introduction

Choline is an essential micronutrient with critical roles in a wide-array of physiologic processes [1]. As a source of methyl groups, choline supports cellular methylation reactions, including genomic methylation, which influences gene expression and DNA stability. Choline also serves as the substrate for the formation of acetylcholine, a neurotransmitter and non-neuronal cell-signaling molecule. Quantitatively, the primary metabolic fate of choline is biosynthesis of phosphatidylcholine (PC), the most abundant phospholipid in cell membranes [2]. Phosphatidylcholine adequacy is critical for cell membrane integrity and the export of fat from the liver by very low density lipoproteins (VLDL) [1]. Choline contributes to PC synthesis through two distinct pathways. Either choline can be phosphorylated, entering the cytidine diphosphate (CDP)-choline pathway where it is converted directly to PC, or, its methyl groups can be used in the triple-methylation of phosphatidylethanolamine (PE) to PC, which occurs by the phosphatidylethanolamine *N*-methyltransferase (PEMT) pathway [3]. This pathway is also responsible for endogenous choline production as PC can be produced through the PEMT pathway using PE and folate or methionine-derived methyl groups in the absence of dietary choline. The efficiency of endogenous choline production varies from person to person, and is greater among pre-menopausal women because PEMT is up regulated by estrogen. However, choline itself is used to support the PEMT pathway and is considered an essential dietary requirement because endogenous production capacity is not enough to support biological choline requirements [4,5,6]. Dietary choline restriction causes acute muscle and liver dysfunction and choline must be obtained from the diet to prevent deficiency [7]. However, there is a large inter-individual variation in dietary choline requirement that depends upon genetic and physiological factors [4].

**Figure 1:**
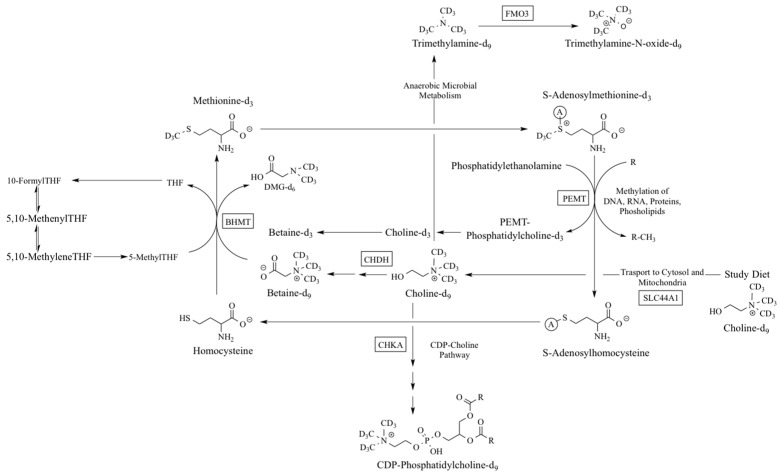
An overview of the metabolic fate of the isotopically labeled choline-d_9_ consumed by study participants. SNPs in squared enzymes were examined. Choline kinase-α (*CHKA*) rs10791957; choline dehydrogenase (*CHDH*) rs9001, *CHDH* rs12676; betaine homocysteine methyltransferase (*BHMT*) rs3733890; phosphatidylethanolamine *N*-methyltransferase (*PEMT*) rs7946, *PEMT* rs4646343; solute carrier 44A1 (*SLC44A1)* rs7873937, *SLC44A1* rs3199966; and flavin monooxygenase isoform 3 (*FMO3)* rs2266782).

Considering the critical metabolic and structural roles of choline, it is not surprising that genetic variants that alter choline metabolism have been linked to increased risk for acute skeletal muscle and liver pathologies under conditions of dietary choline deprivation [8], as well as birth defects and other diseases in the general population (Table 1). However, the underlying metabolic signatures of these variants are not well characterized, and it is unknown whether genotypic differences persist among women meeting choline intake recommendations. Moreover, little is known about the effect of gene–nutrient interactions on choline metabolism and functional outcomes in reproductive states (i.e., pregnancy and lactation) that increase the metabolic use of choline [9]. We have previously shown that genetic variants in folate-metabolizing genes are associated with differences in choline dynamics and partitioning among women meeting current intake recommendations [9]. In the present study, we used isotopically labeled dietary choline to characterize differences in the metabolic flux and partitioning of dietary choline among carriers of genetic polymorphisms in choline metabolizing enzymes (Figure 1).

## 2. Results

### 2.1. Genotype Distribution

The distribution of genotypes within our cohort (*n* = 75) is depicted in Table 2. Because of the relatively low prevalence of the variant allele, heterozygous and homozygous variant individuals were combined to examine the effect of variant allele presence on metabolic outcomes. The number of participants in each sub-group analysis varies by gene and metabolic outcome. For example, if there are no interactions, there are more participants in each group because we are comparing all of the women of a certain genotype against all of the women of the other. On the other hand, when interactions are present, the groups are stratified by the interacting factor (e.g., reproductive status and/or choline intake), which decreases the number of participants per group.

### 2.2. CHKA rs10791957

**Figure 2:**
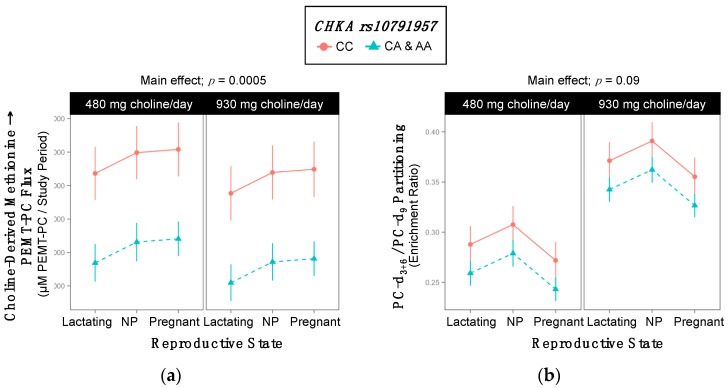
Effect of the *CHKA* rs10791957 variant on the metabolic flux and partitioning of dietary choline. (**a**) Choline-derived methionine → PEMT-PC flux; (**b**) PC-d_3+6_/PC-d_9_ partitioning.

Genotype influenced the production of PEMT-PC and the partitioning of dietary choline between PEMT-PC and CDP-PC (Table 3 and Table S1). Specifically, variant women exhibited a lower turnover of choline-derived methionine → PEMT-PC (8840 ^±291^ vs.11,512 ^± 664^ µM PC/study period; *p* = 0.0005) over the study period and tended to have lower PC-d_3+6_/PC-d_9_ enrichment ratios than non-variants (0.30 ^±0.007^ vs. 0.33 ^±0.015^; *p* = 0.09) (Table 3, Figure 2).

### 2.3. CHDH rs9001

**Figure 3:**
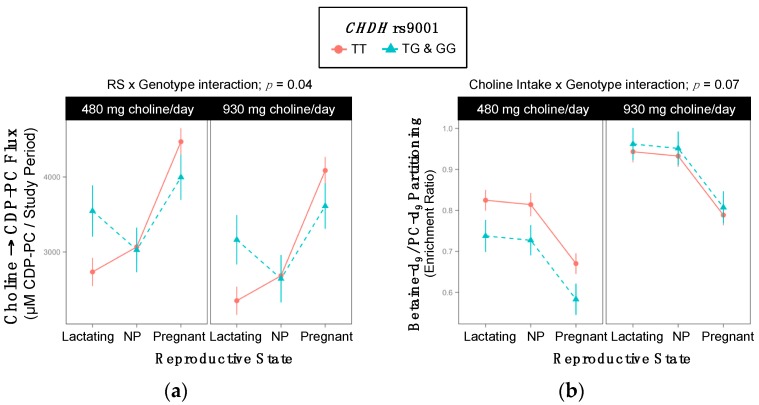
Effect of the *CHDH* rs9001 variant on the metabolic flux and partitioning of dietary choline. (**a**) Choline → CDP-PC flux; (**b**) Betaine-d_9_/PC-d_9_ partitioning.

Genotype interacted with reproductive state to influence the turnover of choline → CDP-PC (*p* = 0.04) (Table 4, Figure 3a). While differences in choline → CDP-PC flux were not observed among pregnant or non-pregnant women, among lactating women, variants tended to exhibit a greater turnover of choline → CDP-PC as compared to non-variants (3355 ^±295^ vs. 2541 ^±167^ µM PC/study period; *p* = 0.09) (Table 4).

In addition, genotype tended to interact with choline intake to predict betaine-d_9_/PC-d_9_ enrichment (*p* = 0.07) (Table 5, Figure 3b). Within the lower choline intake group, variant women exhibited lower betaine-d_9_/PC-d_9_ enrichment ratios than non-variants (0.68 ^±0.03^ vs. 0.77 ^±0.02^; *p* = 0.06) (Table 5). Genotypic differences were not observed among women consuming the higher choline intake.

### 2.4. CHDH rs12676

**Figure 4:**
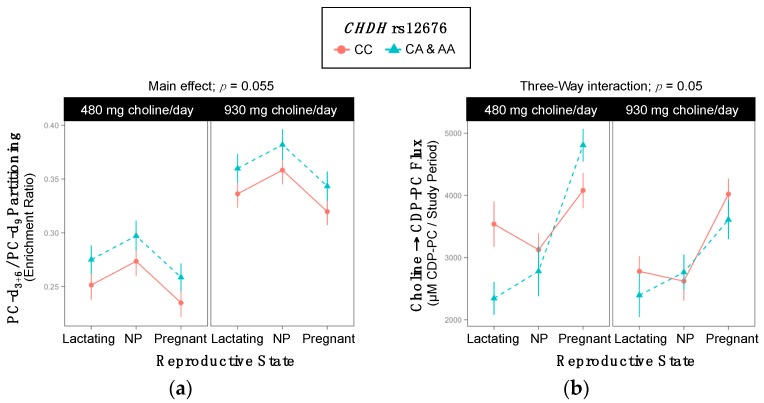

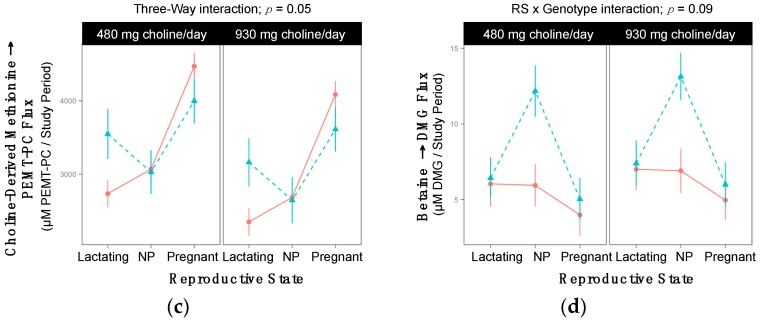
Effect of the *CHDH* rs12676 variant on the metabolic flux and partitioning of dietary choline. (**a**) PC-d_3+6_/PC-d_9_ partitioning; (**b**) Choline → CDP-PC flux; (**c**) Choline-derived methionine → PEMT-PC flux; (**d**) Betaine → DMG flux.

Genotype influenced the partitioning of dietary choline between PEMT-PC and CDP-PC (Figure 4). Specifically, variant individuals tended to have greater PC-d_3+6_/PC-d_9_ enrichment ratios as compared to non-variants (0.32 ^±0.009^ vs. 0.30 ^±0.008^; *p* = 0.055) (Table 6, Figure 4a).

In line with this finding, genotype interacted with reproductive state and choline intake to influence the flux of choline → CDP-PC (*p* = 0.05) and choline-derived methionine → PEMT-PC (*p* = 0.05) (Table S1). The only detectable differences were among lactating women in the lower choline intake group. Within this subset, variants exhibited a lower turnover of both choline → CDP-PC (2345 ^±264^ vs. 3538 ^±364^ µM PC/study period; *p* = 0.06) and choline-derived methionine → PEMT-PC (7182 ^±799^ vs. 12,358 ^±1130^ µM PC/study period; *p* = 0.003) as compared to non-variants (Table 7, Figure 4b,c).

Finally, genotype tended to interact with reproductive state to influence the metabolic flux of betaine → DMG (*p* = 0.09). Non-pregnant variant women exhibited greater turnover of betaine → DMG as compared to non-pregnant non-variant women (6.9 ^±1.3^ vs. 6.5 ^±1.3^ µM DMG/study period; *p* = 0.01) (Table 8, Figure 4d).

### 2.5. BHMT rs3733890

**Figure 5:**
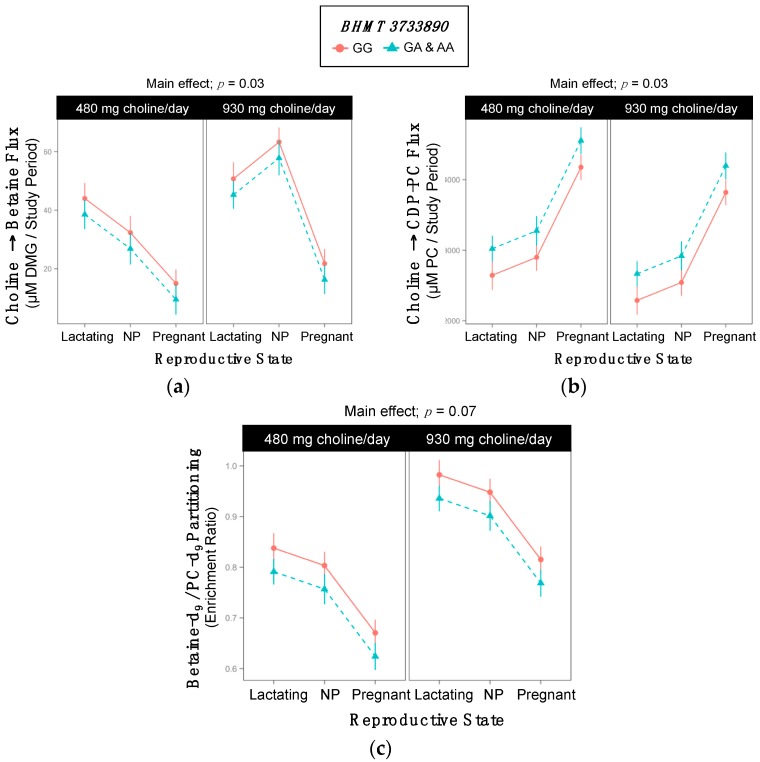
Effect of the *BHMT* rs3733890 variant on the metabolic flux and partitioning of dietary choline. (**a**) Choline → betaine flux; (**b**) Choline → CDP-PC flux; (**c)** Betaine-d_9_/PC-d_9_ partitioning.

Genotype influenced the metabolic flux of choline → betaine (*p* = 0.03) and choline → CDP-PC (*p* = 0.03) and accordingly, the partitioning of dietary choline between betaine and CDP-PC (*p* = 0.07) (Table S1, Figure 5). Variant women exhibited a greater turnover of choline → CDP-PC (3440 ^±122^ vs. 3063 ^±122^ µM PC/study period; *p* = 0.03) and a non-significantly lower turnover of choline → betaine (32 ^±3^ vs. 38 ^±3^ µM betaine/study period; *p* = 0.2) (Although the model effect of genotype was significant for the metabolic flux of choline → betaine, the comparison between the two genotypes was not, the difference being that the model effect does not account for the presence of a reproductive state by choline intake interaction.) (Table 9, Figure 5a,b). Consistent with these findings, variant individuals tended to have lower betaine-d_9_/PC-d_9_ enrichment ratios as compared to non-variants (0.77 ^±0.02^ vs. 0.82 ^±0.02^; *p* = 0.07) (Table 9, Figure 5c).

### 2.6. PEMT rs4646343

**Figure 6:**
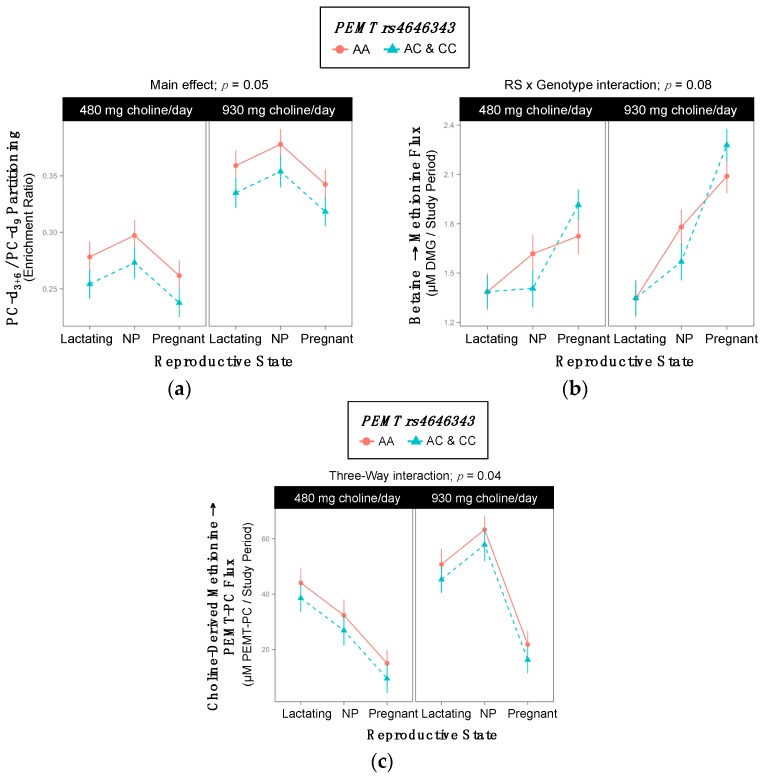
Effect of the *PEMT* rs4646343 variant on the metabolic flux and partitioning of dietary choline. (**a**) PC-d_3+6_/PC-d_9_ partitioning; (**b**) Betaine → methionine flux; (**c**) Choline-derived methionine → PEMT-PC flux.

Genotype influenced partitioning of dietary choline between PEMT-PC and CDP-PC (Table S1, Figure 6). Women with the variant allele exhibited lower PC-d_3+6_/PC-d_9_ enrichment ratios as compared to women without (0.30 ^±0.008^ vs. 0.32 ^±0.009^; *p* = 0.05) (Table 10, Figure 6a).

Genotype also interacted with reproductive state to influence the metabolic flux of betaine → methionine (*p* = 0.08), though individual effects of genotype were not detectable after stratifying by reproductive state. Finally, genotype interacted with choline intake and reproductive state to influence the metabolic flux of choline-derived methionine → PEMT-PC (*p* = 0.04), though individual effects of genotype were not detectable after stratifying by choline intake (Figure 6b,c).

### 2.7. PEMT rs7946

**Figure 7:**
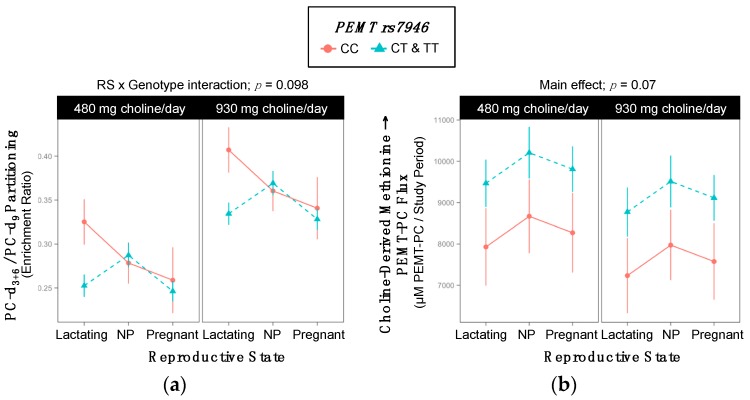
Effect of the *PEMT* rs7946 variant on the metabolic flux and partitioning of dietary choline. (**a**) PC-d_3+6_/PC-d_9_ partitioning; (**b**) Choline-derived methionine → PEMT-PC flux.

Genotype interacted with reproductive state to influence the partitioning of dietary choline between PEMT-PC and CDP-PC (*p* = 0.097) (Table S1, Figure 7a). While genotypic differences in PC-d_3+6_/PC-d_9_ enrichment ratios were not observed among pregnant and non-pregnant women, lactating variant women exhibited lower PC-d_3+6_/PC-d_9_ enrichment ratios as compared to lactating non-variant women (0.29 ^±0.01^ vs. 0.37 ^±0.03^; *p* = 0.03) (Table 11).

In addition, genotype influenced the metabolic flux of choline-derived methionine → PEMT-PC (Table S1, Figure 7b). Variant women had non-significantly greater turnover of choline-derived methionine → PEMT-PC as compared to non-variants (9481 ^±312^ vs. 7942 ^±765^ µM PC/study period; *p* = 0.07) (Table 12).

### 2.8. FMO3 rs2266782

**Figure 8:**
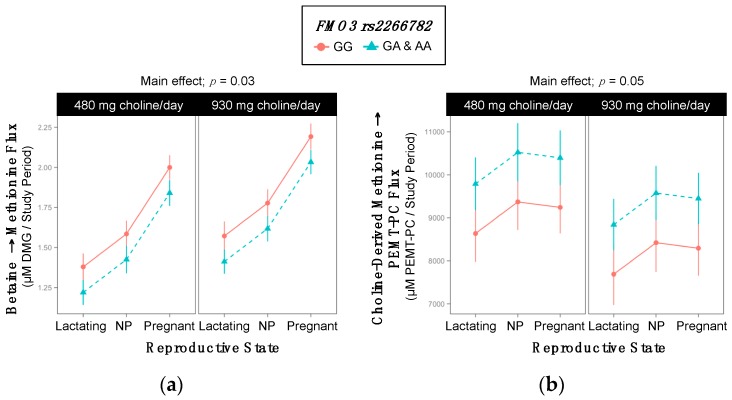
Effect of the *FMO3* rs3733890 variant on the metabolic flux and partitioning of dietary choline. (**a**) Betaine → methionine flux; (**b**) Choline-derived methionine → PEMT-PC flux.

Variant women had greater turnover of betaine → methionine (1.6 ^±0.05^ vs. 1.8 ^±0.05^ µM methionine/study period; *p* = 0.03) and a greater turnover of choline-derived methionine → PEMT-PC (9761 ^±384^ vs. 8609 ^±433^ µM PC/study period; *p* = 0.05) as compared to non-variants (Table 13, Figure 8).

### 2.9. SLC44A1 rs7873937

**Figure 9:**
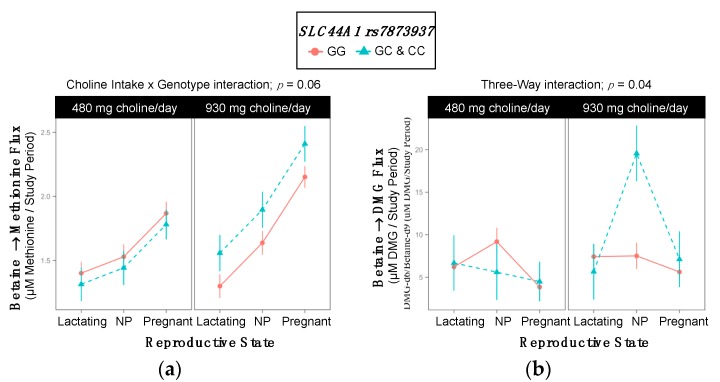

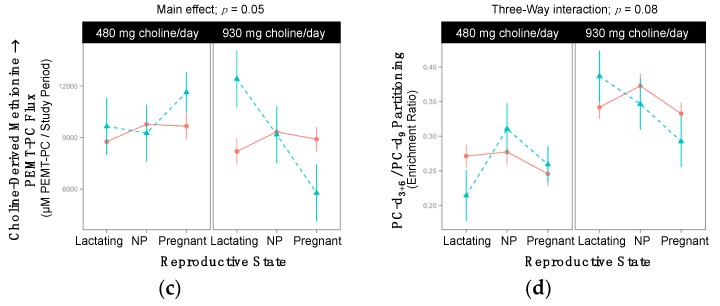
Effect of the *SLC44A1* rs7873937 variant on the metabolic flux and partitioning of dietary choline. (**a**) Betaine → methionine flux; (**b**) Betaine → DMG flux; (**c**) Choline-derived methionine → PEMT-PC flux; (**d**) PC-d_3+6_/PC-d_9_ partitioning.

Genotype interacted with choline intake to influence the turnover of betaine → methionine (*p* = 0.06) (Table S1, Figure 9a). Although non-variants did not exhibit differences in the turnover of betaine → methionine as a function of choline intake, variant women exhibited greater betaine → methionine turnover in the higher choline intake group as compared to the lower intake group (1.95 ^±0.11^ vs. 1.51 ^±0.12^ µM methionine/study period; *p* = 0.03) (Table 14).

In addition, genotype interacted with reproductive state and choline intake to influence the metabolic flux of betaine → DMG (*p* = 0.04) (Table S1, Figure 9b). While genotypic differences were not observed in the lower choline intake group, within the higher intake group, non-pregnant variant women exhibited a greater turnover of betaine → DMG (19.6 ^±3.3^ vs. 7.5 ^±1.5^ µM DMG/study period; *p* = 0.02) (Table 15). Furthermore, similar to betaine → methionine turnover, although non-variants did not display differences in betaine → DMG as a function of choline intake (*p* > 0.99), variant non-pregnant women exhibited increased betaine → DMG in the higher choline intake group as compared to the lower intake group (19.6 ^±3.3^ vs. 5.6 ^±3.3^ µM DMG/study period; *p* = 0.05) (Table 16).

Genotype also interacted with reproductive state and choline intake to influence the metabolic flux of choline-derived methionine → PEMT-PC (*p* = 0.05) (Table S1 and Figure 9c). Genotypic differences were not detected within intake groups, however, variant and non-variant women responded differently to increased choline intake in a manner that depended upon reproductive state (Table 16). Specifically, only pregnant women with the variant exhibited different metabolic flux of choline-derived methionine → PEMT-PC between intake groups with lower flux in the higher intake group as compared to the lower intake group (5780 ± 1658 vs. 11635 ± 1172 µM PEMT-PC/study period; *p* = 0.07).

Finally, genotype interacted with reproductive state and choline intake to influence the partitioning of dietary choline between PEMT-PC and CDP-PC (*p* = 0.08) (Figure 9d). Genotypic differences were not detected within intake groups; however, variant and non-variant women responded differently to increased choline intake in a manner that depended upon reproductive state (Table 16). Specifically, across reproductive states, women without the variant exhibited greater PC-d_3+6_/PC-d_9_ enrichment ratios in the higher choline intake group as compared to the lower (*p* ≤ 0.05). Among women with the variant however, only lactating women exhibited increased PC-d_3+6_/PC-d_9_ enrichment ratios in the higher choline intake group as compared to the lower (*p* = 0.02) (Table 16).

### 2.10. SLC44A1 rs3199966

**Figure 10:**
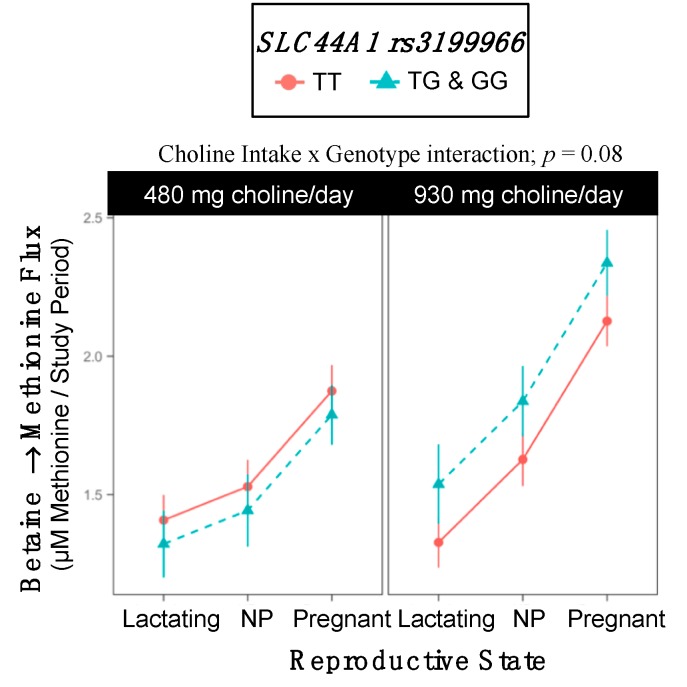
Effect of the *SLC44A1* rs3199966 variant on the metabolic flux and partitioning of dietary choline.

Genotype interacted with choline intake to influence the turnover of betaine → methionine (*p* = 0.08). While non-variants did not exhibit differences in the turnover of betaine → methionine as a function of choline intake, variant women exhibited greater betaine → methionine turnover in the higher choline intake group as compared to the lower intake group (1.90 ^±0.11^ vs. 1.52 ^±0.096^ µM methionine/study period; *p* = 0.04) (Table 17, Figure 10).

## 3. Discussion

These results demonstrate that common genetic variants in choline metabolizing genes alter the metabolic signature of choline in three ways: (i) the use of dietary choline as a methyl donor (*CHKA* rs10791957, *CHDH* rs9001, *CHDH* rs12676, *PEMT* rs4646343, *PEMT* rs7946, *FMO3* rs2266782, *SLC44A1* rs7873937 and *SLC44A1* rs3199966); (ii) the partitioning of dietary choline between betaine and CDP-PC synthesis (*CHDH* rs9001 and *BHMT* rs3733890); and (iii) the distribution of dietary choline between PEMT-PC and CDP-PC (*CHKA* rs10791957, *CHDH* rs12676, *PEMT* rs4646343, *PEMT* rs7946 and *SLC44A1* rs7873937). Such metabolic differences may contribute to disease pathogenesis and prognosis over the long-term.

### 3.1. CHKA (dbSNP: rs10791957; NC_000011.10: g.68100081 C > A)

Choline kinase-α catalyzes the cytosolic phosphorylation of choline to phosphocholine, which comprises the first step of the CDP-PC pathway. The rs10791957 SNP is located in the first intron, a possible enhancer region, and is associated with a decreased risk of organ dysfunction in women deprived of choline, as well as a decreased risk of type 2 diabetes [10,11]. Although the rate-limiting step in the CDP-PC pathway is considered to be the nucleotidyl transfer of CDP to phosphocholine, catalyzed by phosphocholine cytidylytransferase, differences in *CHKA* expression influence cellular PC production [23]. *CHKA* expression contributes to the regulation of cellular proliferation and apoptosis and *CHKA* overexpression and phosphocholine accumulation are associated with increased proliferation and oncogenesis [24,25]. Furthermore, tissue-specific *CHKA* expression (modulated in part by the circadian clock), has been proposed as a possible regulatory mechanism for the CDP-PC pathway [26]. Our data support a role for the *CHKA rs10791957* variant as an additional factor that may modulate PC homeostasis. Specifically, the variant appears to decrease the use of dietary choline for PEMT-PC synthesis relative to CDP-PC synthesis. Variant individuals displayed decreased turnover of choline-derived methionine → PEMT-PC over the study period, indicating decreased activity of PEMT relative to women without the variant, and also tended to exhibit lower relative PEMT-PC/CDP-PC enrichment as compared to non-variants. These differences may be direct (arising from altered CHKA activity) or indirect (arising from changes in downstream signaling that regulate phospholipid metabolism) consequences of differences in *CHKA* expression. Notably, the decreased PEMT activity observed among variant women may provide a metabolic basis for the decreased risk of type 2 diabetes among variant individuals given that PEMT knockout mice are protected from high-fat diet induced obesity and insulin resistance (though not protected from hepatic steatosis) [27]. Additional studies are needed to determine whether *CHKA rs10791957* genotype distribution influences the relationship between diet, obesity and insulin resistance.

### 3.2. CHDH (dbSNP: rs9001; c.119 A > C; p.Glu40Ala) and (dbSNP: rs12676; c.233T > G; p.Leu78Arg)

CHDH is a flavin-dependent, mitochondrial enzyme that oxidizes choline to betaine aldehyde. The *CHDH* rs9001 variant is associated with a decreased risk of choline deficiency, while the rs12676 variant is associated with an increased risk among pre-menopausal women, which suggests opposing effects of these variants on CHDH activity [28].

Our results suggest that women with the rs9001 variant partition dietary choline to the CDP-choline pathway at the expense of betaine synthesis. Specifically, rs9001 variant lactating women exhibited increased turnover of choline → CDP-PC as well as non-significantly decreased betaine-d_9_/PC-d_9_ enrichment ratios in the lower intake group (Figure 3). They also exhibited slightly and non-significantly lower PC-d_3+6/_PC-d_9_ enrichment ratios (0.29 ^± 0.01^ vs. 0.31 ^± 0.01^; *p* = 0.1), further supporting the notion that women (particularly lactating women) with the rs9001 variant may relatively favor CDP-PC synthesis over PEMT-PC synthesis. This relatively greater use of choline for CDP-PC synthesis as compared to PEMT synthesis among CHDH rs9001 variants may conserve choline stores. For example, while each unit of choline directed to the CDP-choline pathway yields one unit of PC, choline converted to betaine (and eventually SAM) has many metabolic fates besides PEMT-PC production and requires three choline-derived methyl groups to yield just one additional unit of PC. In direct contrast, *CHDH* rs12676 variant women appear to favor the use of dietary choline for PEMT-PC synthesis relative to CDP-PC. rs12676 variant women exhibited higher PC-d_3+6/_PC-d_9_ enrichment ratios, non-pregnant rs12676 variant women exhibited a greater use of choline as a methyl donor, and lactating rs12676 variant women exhibited greater use of choline for PEMT-PC synthesis and lower use of choline for CDP-PC synthesis within the study period as compared to non-variants (Figure 4).

Importantly, our findings identify opposite metabolic differences for these variants, which is consistent with a decreased and increased risk of choline deficiency, respectively as previously reported by others [10,14]. In some ways, however, our data appear to contrast previous work, possibly due to a different effect of these variants by tissue, sex (the *CHDH* gene is under the control of an estrogen promoter), diet and other environmental factors [29]. Specifically, the rs12676 variant confers a relative loss of function in male sperm as variant men exhibit decreased sperm ATP and dyspmorphic mitochondrial structures similar to *Chdh*^−/−^ mice, as well as decreased *CHDH* protein in sperm [14]. The mechanism however is unclear and it is unknown whether this effect is due to increased expression of *CHDH* or decreased degradation. Additionally, a previous study identified increased dimethyl-arsenic:mono-methyl arsenic in *CHDH* rs9001 variant individuals exposed to arsenic, indicating increased efficiency of arsenic methylation [12]. The authors postulated that this increased efficiency of arsenic methylation (which facilitates detoxification) among rs9001 variant individuals may be due to a gain of function in *CHDH* activity that increases the conversion of choline to betaine, increasing SAM availability. Another possible interpretation is that the variant comprises a loss of *CHDH* activity. Decreased conversion of choline to betaine might increase the availability of choline for CDP-PC synthesis, reducing the burden on SAM for PC synthesis (PEMT is one of the main consumers of SAM in the liver), and increasing the availability of SAM for arsenic methylation. Overall, our results highlight that the *CHDH* rs9001 and *CHDH* rs12676 SNPs exert opposing metabolic effects, not only among individuals deprived of choline or exposed to one-carbon stressing conditions, but also among healthy women consuming choline intakes relevant to the general population.

### 3.3. BHMT (dbSNP: rs3733890; c.716 G > A, also Known as c.742 G > A; p.Arg239Gln)

Betaine homocysteine methyltransferases (BHMT) is a zinc-dependent enzyme that uses betaine to remethylate homocysteine to methionine. It acts primarily in the liver, but is also present in the kidney and optic lens. The *BHMT* rs3733890 variant encodes an arginine to glutamine change at amino acid 239, which results in a lower *K*_m_ (roughly half) for both betaine and homocysteine [30]. The *BHMT* rs3733890 polymorphism has been associated with reduced all-cause mortality in breast cancer patients and a number of developmental outcomes including a decreased risk of orofacial cleft and an increased risk of spina bifida, particularly with high maternal folic acid intake, but results have been mixed [15,16,17,18,31]. There is some evidence that the effect of this variant is modulated by *MTHFR* rs1801133 variant allele presence, however the relationship remains unresolved [18]. Given the scarcity of *MTHFR* rs1801133 and *BHMT* rs3733890 homozygous variant individuals in our cohort, this study was unable to examine this interaction. When evaluated independently, the *BHMT* variant allele was associated with non-significantly decreased turnover of choline → betaine, and increased turnover of choline → CDP-PC as well as a (non-significantly, *p* = 0.07) lower betaine-d_9_/PC-d_9_ enrichment ratio. Together, these results indicate that the variant favors the use of dietary choline for CDP-PC synthesis at the expense of betaine synthesis. These findings can be understood in the context of the effect of this SNP on enzyme kinetics. A lower K_m_ for both betaine and homocysteine, results in increased affinity of the enzyme for these substrates, meaning less betaine is needed among variants to maintain maximal BHMT activity. Partitioning away from betaine, therefore, may conserve dietary choline among variant individuals.

### 3.4. PEMT (dbSNP: rs4646343; c.2768 C > A REV) and (dbSNP: rs7946; c.5465 G > A REV; p.Val175Met)

Phosphatidylethanolamine *N*-methyltransferase (PEMT) catalyzes the de novo synthesis of choline via the triple methylation of PE to form PC [32]. The *PEMT* rs7946 variant encodes a valine to methionine substitution at amino acid 175, which results in decreased enzymatic activity in vitro and may increase susceptibility to non-alcoholic fatty liver disease (NAFLD) [20,21]. Previous work has found a 92% overlap of the intronic *PEMT* rs4646343 SNP with the functional rs12325817 SNP, which is located in the promoter region of the *PEMT* gene, near the estrogen response element, and impedes its estrogen-mediated up-regulation [33]. This impairment leads to an increased susceptibility to organ dysfunction in variant individuals [10]. Although we did not observe direct indications of decreased PEMT activity, the observed decreased PEMT-PC/CDP-PC in rs4646343 variant individuals is consistent with decreased PEMT activity and an impaired estrogen response among variant individuals. Decreased PEMT-PC/CDP-PC was also observed in *PEMT* rs7946 variant lactating women.

Overall, these data suggest a relatively decreased contribution of PEMT-PC relative to CDP-PC in PC pools with both *PEMT* rs4646343 and rs7946 variants. Impaired PEMT activity may compromise PC-DHA supply to extra-hepatic tissue including vital reproductive organs during pregnancy and lactation [34]. Therefore, given that these data support the notion that both PEMT variants lead to functional changes in PC homeostasis at choline intakes meeting current recommendations, these SNPs deserve further study to determine whether such effects are associated with negative clinical outcomes among the general population, whose intakes are well below current recommendations [35].

### 3.5. FMO3 (dbSNP: rs2266782; c.472 G > A; p.Glu158Lys)

FMO3 is a (largely) hepatic enzyme that converts trimethylamine, a breakdown product of choline produced by anaerobic intestinal microbiota, to trimethylamine *N-*oxide (TMAO) [36]. The rs2266782 SNP is a common polymorphism that encodes a glutamate to lysine amino acid change (E158K) in flavin monooxygenase isoform 3 (FMO3) [37]. This variant is associated with a relative loss-of-function and, when in *cis* with other common variants, can cause mild trimethylaminuria (due to a relative excess of trimethylamine), which has largely unknown metabolic consequences [22]. FMO3 is activated by insulin, and knockout in insulin resistant mice prevents hyperglycemia, hyperlipidemia, and atherosclerosis [38]. FMO3 is suppressed by testosterone and up regulated by bile acids, which also stimulate hepatic cholesterol absorption [39].

Differences in TMAO metabolism are known to alter cholesterol transport and influence risk for cardiovascular disease [40,41]. TMAO supplementation in mice has been shown to increase macrophage cholesterol accumulation, which subsequently increases risk for atherosclerosis [41]. More recently, Brown and colleagues identified FMO3 itself (rather than TMAO) as a direct regulator of cholesterol balance, lipid metabolism, and inflammation in mice. FMO3 knockdowns had decreased hepatic cholesterol production, decreased intestinal absorption, and increased hepatic inflammation along with activation of liver X receptor (LXR)-stimulated macrophage reverse cholesterol transport [42]. While a previous study from our group suggested that the variant might be associated with increased use of choline as a methyl donor in men (based on increased DMG pool size) [43], results from the present study, indicate that women with the variant actually use choline *less* as methyl donor. Variant women tended to have a lower turnover of betaine → methionine over the study period. In addition, variant women exhibited a greater turnover of choline-derived methionine → PEMT-PC over the study period, which is consistent with previous findings from our lab that have identified lower methionine excretion among variant individuals (i.e., a greater use of methionine may reduce excretion) [43]. While it is not clear how these findings relate to data in mice, our results strengthen previous evidence of a relationship between FMO3 and phospholipid metabolism and demonstrate that this SNP exerts an effect on the metabolic use of dietary choline.

### 3.6. SLC44A1 (dbSNP: rs7873937; NC_000009.11:g.108089321 G > C) and SLC44A1 (dbSNP: rs3199966; c.1930 T > G; p.Ser644Ala)

Solute Carrier 44A1 (SLC44A1), also referred to as choline transporter-like protein 1 (CTL1), is a transmembrane protein that mediates choline transport across the mitochondrial and plasma membranes [44,45,46]. It is expressed in four major splice variants throughout the brain and central nervous system including the spinal cord, motor neurons, and oligodendrocytes during and after myelination, as well as in the colon and lung. While the majority of people present with liver dysfunction in response to choline deprivation, Zeisel and colleagues noted that some individuals present first with muscle dysfunction, and they tend to carry mutations in the *SLC44A1* gene [10]. Though not exonic, the *SLC44A1* rs7873937 variant allele is associated with an increased susceptibility to muscle dysfunction in humans deprived of choline [10]. *SLC44A1* rs7873937 may exist in a regulatory region that responds to choline status, or may be in linkage disequilibrium with a functional SNP. The rs3199966 polymorphism confers a serine to alanine amino acid substitution, and like rs7873937, is associated with increased susceptibility to muscle dysfunction in humans deprived of choline [10]. For each of these SNPs, rs7873937 and rs3199966, we identified an interaction between genotype and choline intake that governed the use of choline as a methyl donor. The finding that the effect of genotype depends on choline intake aligns with in vitro evidence that dietary choline is known to modulate expression of the *SLC44A1* gene [47]. Specifically, for both SNPs, variant (but not non-variant) individuals exhibited greater turnover of betaine → methionine in the higher choline intake group as compared to the lower, suggesting that additional choline was used for methyl donation. For *SLC44A1* rs7873937, this effect was partially mirrored in a greater turnover of betaine → DMG at the higher choline intake among non-pregnant women. While we expect that betaine → DMG and betaine → methionine turnover would be identical, our results are not exactly the same, and this is likely due to differences in choline partitioning and sequestration throughout the various pools in the body.

### 3.7. Study Limitations

Given the post-hoc design, which did not preemptively evenly distribute participants across reproductive states, choline intake groups, and genotypes, some comparisons that involved two and three way interactions ended up with small sample sizes. Other limitations include possible unequal distribution of other functional variants within the study population, and the fact that circulating metabolites may not be in equilibrium with all tissues. Nonetheless, the present study benefitted from a highly controlled study environment and the precision afforded from the use of an isotopic tracer.

### 3.8. Conclusions

In sum, these data provide compelling evidence that common SNPs modulate choline partitioning in women of reproductive age consuming intakes that are relevant to the population at large. These metabolic differences may contribute to disease pathogenesis and prognosis over the long-term given relationships between methyl group and PC homeostasis and disease. Therefore, these SNPs deserve further study in a clinical and epidemiological context.

## 4. Materials and Methods

### 4.1. Participants and Study Design

This study was a follow-up investigation of a long-term randomized controlled feeding study conducted among women of reproductive age by Yan et al. [6]. Healthy third-trimester pregnant (*n* = 26), lactating (*n* = 28), and non-pregnant (*n* = 21), women consumed the study diet containing 380 mg choline/day, and either 100 or 550 mg choline/day from supplemental choline chloride (Balchem) for 10–12 weeks [6]. All participants also consumed a daily prenatal multivitamin (Pregnancy Plus; Fairhaven Health, LLC, Bellingham, WA, USA) containing 600 µg folic acid, a daily docasahexanoic acid supplement (200 mg, Neuromins; Nature’s Way, Perris, CA, USA), and a potassium and magnesium supplement (General Nutrition Corp, Pittsburgh, PA, USA) thrice weekly. Beginning at week 6, participants consumed 22% of their total choline in the form of choline chloride-(trimethyl-d_9_) (Cambridge Isotope Laboratories, Tewksbury, MA, USA, 98%). Fasting blood (10 h) was collected at study baseline and throughout the study and processed as previously described [6]. All samples were stored at −80 °C until analysis. The study was approved by the Institutional Review Boards at Cornell University and Cayuga Medical Center and was registered at clinicaltrials.gov as NCT01127022. All participants provided informed consent.

### 4.2. Genotyping

DNA was extracted from buffy coat for genotyping of *PEMT* rs7946, *PEMT* rs4646343, *CHDH* rs9001, *CHDH* rs12676, *CHKA* rs10791957, *SLC44A1* rs7873937, *SLC44A1* rs3199966, *BHMT* rs3733890, and *FMO3* rs2266782 SNPs using the Qiagen DNeasy Blood and Tissue on a LightCycler480 (Roche, Indianapolis, IN, USA). Endpoint genotyping was carried out as previously described using participant DNA and two commercially available products, (Applied Biosystems TaqMan Genotyping Master Mix and Thermo Fisher Scientific Assay Mix, Waltham, MA, USA) on a LightCycler 480 (Roche) in our facility [9].

### 4.3. Enrichment of Choline Metabolites

Choline metabolites were extracted from blood and enrichments of choline-d_9_, choline-d_3_, betaine-d_9_, betaine-d_3_, and DMG-d_6_ as well as PC-d_3_, PC-d_6_, and PC-d_9_, were measured using a TSQ Quantum Access triple quadrupole LCMS system (Thermo) operated in positive-ion mode using electrospray ionization as previously described in detail [9]. Enrichments of methionine-d_3_ were measured by gas chromatography-mass spectrometry (GC-MS) [9]. Enrichment percentages were calculated by dividing the area of each isotopically labeled choline metabolite by the total area of all isotopomers and multiplying by 100% (Equation (1)).

(1) $$Enrichment_{metabolite}=\frac{labeled\;metabolite\times 100\%}{labeled+unlabeled\;metabolite}$$

### 4.4. Statistical Analysis

Seven metabolic outcomes in plasma were examined as primary response variables. Two of these outcomes were chosen to reflect partitioning between metabolic pathways (enrichment ratios of betaine-d_9_/PC-d_9_ and PC-d_3+6_/PC-d_9_). The other five outcomes were chosen to reflect flux through metabolic pathways and included turnover of choline → betaine, choline → CDP-PC, betaine → DMG, betaine → methionine, and choline-derived methionine → PEMT-PC within the study period. Metabolic flux was defined as the rate of turnover of metabolic precursors → products in µmols/L/study period over the three-week period of label exposure (Equation (2)), where $Enrichment_{Product}$ and $Enrichment_{Precursor}$ are enrichments (percentages) of the product and precursor, and $Pool\;Size_{Product}$ is the plasma pool size in µmoles of metabolite product per liter of plasma.

(2) $$Rate_{turnover}=\frac{Enrichment_{Product}\times Pool\;Size_{Product}}{Enrichment_{Precursor}}$$

The effect of genetic variation on each of these seven outcomes as a function of SNP genotype was assessed using linear models. Due to limited variant allele presence within our sample (Table 1), heterozygous and homozygous variant individuals were grouped together to examine the effect of variant allele presence. Reproductive status, choline intake group (480 or 930 mg choline/day), and possible interactions were included as covariates. A backwards selection was used in which BMI was retained at an α-cutoff of 0.05 and interactions were retained at an α-cutoff of 0.1 (the higher interaction cutoff was selected to prevent the interpretation of main effects in the presence of interactions). Model assumptions and the fit of the model to the data were assessed with standard diagnostic methods. Two lactating participants with choline-d_9_ enrichment values greater than 2 standard deviations from the mean were excluded from the entire analysis. All statistical analysis was performed using the lsmeans package in the R statistical programming environment, available from CRAN 2014 [48]. Data are presented as predicted least-squared means, unless otherwise noted. Reported *p*-values include Bonferroni corrections for multiple comparisons and were considered significant at an an α-cutoff of 0.05.

## Figures and Tables

**Table 1:** Summary of examined SNPs and their connections to disease.

Gene	Function	SNP	Choline Deficiency Risk	Disease Associations	References
*CHKA*	Phosphorylates choline, first step in CDP-choline pathway	rs10791957	↓ risk organ dysfunction *	↓ risk type 2 diabetes	[10,11]
*CHDH*	First step in oxidation of choline to betaine	rs9001	↓ risk organ dysfunction	↑ arsenic methylation	[10,12]
		rs12676	↑ risk organ dysfunction **	↑ breast cancer risk	[13,14]
				↓ sperm ATP and altered sperm motility	
*BHMT*	Converts homocysteine to methionine using betaine as a methyl donor	rs3733890	-	↓ breast cancer mortality	[10,15,16,17,18]
				↑ orofacial cleft	
				↑ spina bifida (mixed results)	
*PEMT*	Uses SAM to triply methylate PE to form PC (endogenous choline synthesis)	rs4646343	↑ risk organ dysfunction	↑ PEMT expression in adipose	[19]
				↑ waist to hip ratio	
		rs7946	-	↑ Non-alcoholic fatty liver disease	[20,21]
*FMO3*	Converts TMA to TMAO	rs2266782	-	↑ trimethylaminuria	[22]
*SLC44A1*	Transports choline across the cellular and mitochondrial membranes	rs7873937	↑ risk muscle damage		[10]
		rs3199966	↑ risk muscle damage		[10]

* Among women; ** among pre-menopausal women. ↑ increased, ↓ decreased.

**Table 2:** Genotype distribution (# of participants) among reproductive states and choline intake groups.

Group	480 mg Choline/day			930 mg Choline/day		
# Of Variant Alleles	0	1	2	0	1	2
*CHKA* rs10791957						
Lactating	2	5	6	2	4	7
Non-pregnant	2	5	3	2	7	2
Pregnant	2	6	5	1	3	9
*CHDH* rs9001						
Lactating	11	1	1	10	2	1
Non-pregnant	6	4	0	9	2	0
Pregnant	10	2	1	10	2	1
*CHDH* rs12676						
Lactating	5	7	1	9	3	1
Non-pregnant	7	1	2	5	6	0
Pregnant	6	6	1	8	4	1
*BHMT* rs3733890						
Lactating	5	6	2	3	8	2
Non-pregnant	4	6	0	9	1	1
Pregnant	8	4	1	6	6	1
*PEMT* rs4646343						
Lactating	6	4	3	6	6	1
Non-pregnant	5	5	0	6	4	1
Pregnant	5	5	3	6	4	3
*PEMT* rs7946						
Lactating	2	2	9	2	2	9
Non-pregnant	2	5	3	3	5	3
Pregnant	0	6	7	2	3	8
*FMO3* rs2266782						
Lactating	7	5	1	3	8	2
Non-pregnant	6	3	1	4	4	3
Pregnant	6	4	3	7	4	1
*SLC44A1* rs7873937						
Lactating	11	2	0	11	2	0
Non-pregnant	8	2	0	9	2	0
Pregnant	9	3	1	11	2	0
*SLC44A1* rs3199966						
Lactating	10	3	0	11	2	0
Non-pregnant	8	2	0	8	3	0
Pregnant	8	4	1	9	4	0

**Table 3:** *CHKA* rs10791957 genotype alters plasma choline metabolite partitioning and flux. Values are least-squared means ± standard errors for each group. PC-d_3+6_/PC-d_9_ values are ratios, choline-derived methionine → PEMT-PC values are in µM PEMT-PC/study period.

Metabolic Outcome	WT	Variant	*p*-Value
**Choline-Derived Methionine → PEMT-PC**	11,512 ^±664^	8840 ^±291^	0.0005
**PC-d_3+6_/PC-d_9_**	0.33 ^±0.015^	0.30 ^±0.007^	0.09

**Table 4:** Comparison of choline → CDP-PC turnover between genotypes by reproductive state. Values are least-squared means ± standard errors in ratios.

Metabolic Outcome and Group	WT	Variant	*p*-Value
**Choline → CDP-PC**			
**RS × Gene Interaction; *p* = 0.04**			
Lactating	2542 ^±167^	3355 ^±324^	0.09
Non-pregnant	2875 ^±185^	2836 ^±295^	>0.99
Pregnant	4279 ^±^^159^	3804 ^±^^290^	0.5

**Table 5:** Comparison of betaine-d_9_/PC-d_9_ enrichment ratios between *CHDH* rs9001 genotypes by choline intake group. Values are least-squared means ± standard errors in ratios.

Metabolic Outcome and Group	WT	Variant	*p*-Value
**Betaine-d_9_/PC-d_9_**			
**Cho × Gene Interaction; *p* = 0.07**			
480 mg Choline/day	0.77 ^±0.02^	0.68 ^±0.03^	0.06
930 mg Choline/day	0.89 ^±0.02^	0.91 ^±0.04^	>0.99

**Table 6:** *CHDH* rs12676 genotype alters plasma choline metabolite partitioning. Values are least-squared means ± standard errors for each group. PC-d3+9/PC-d9 values are ratios.

Metabolic Outcome	WT	Variant	*p*-Value
**PC-d_3+6_/PC-d_9_**	0.30 ^±0.008^	0.32 ^±0.009^	0.055

**Table 7:** *CHDH* rs12676 genotype alters the metabolic flux of plasma choline metabolites. Values are least-squared means ± standard errors. Choline → CDP-PC and choline-derived methionine → PEMT-PC values are in µM PEMT-PC/study period. *p*-values represent the highest order interaction or main effect and pairwise comparisons between genotypes within intake groups.

Metabolic Outcome	480 mg Choline/day WT	480 mg Choline/day Variant	*p*-Value	930 mg Choline/day WT	930 mg Choline/day Variant	*p*-Value
**Choline → CDP-PC 3-Way Interaction; *p* = 0.05**						
Lactating	3538 ^±364^	2345 ^±264^	0.06	2779 ^±246^	2394 ^±350^	>0.99
Non-pregnant	3127 ^±266^	2782 ^±403^	>0.99	2621 ^±312^	2764 ^±285^	>0.99
Pregnant	4080 ^±285^	4809 ^±263^	0.39	4021 ^±248^	3609 ^±313^	>0.99
**Choline-Derived Methionine → PEMT-PC 3-Way Interaction; *p* = 0.05**						
Lactating	12358 ^±1130^	7182 ^±799^	0.003	9151 ^±799^	8467 ^±1304^	>0.99
Non-pregnant	9334 ^±854^	10451 ^±1304^	>0.99	9338 ^±1010^	9272 ^±922^	>0.99
Pregnant	9668 ^±922^	10782 ^±854^	>0.99	8728 ^±799^	7929 ^±1010^	>0.99

**Table 8:** Comparison of choline → CDP-PC turnover between *CHDH* rs12676 genotype by reproductive state. Values are least-squared means ± standard errors in ratios.

Metabolic Outcome and Group	WT	Variant	*p*-Value
**Betaine → DMG RS × Gene Interaction; *p* = 0.09**			
Lactating	6.5 ^±1.3^	6.9 ^±1.3^	>0.99
Non-pregnant	6.4 ^±1.3^	12.7 ^±1.5^	0.01
Pregnant	4.5 ^±1.2^	5.5 ^±1.3^	>0.99

**Table 9:** *BHMT* rs3733890 genotype alters plasma choline metabolite partitioning and flux. Values are least-squared means ± standard errors for each group. Betaine-d9/PC-d9 values are ratios, choline → betaine values are in µM betaine/study period and choline → CDP-PC values are in µM PC/study period.

Metabolic Outcome	WT	Variant	*p*-Value
**Choline → Betaine**	38 ^±3^	32 ^±3^	0.2
**Choline → CDP-PC**	3063 ^±122^	3440 ^±122^	0.03
**Betaine-d_9_/PC-d_9_**	0.82 ^±0.02^	0.77 ^±0.02^	0.07

**Table 10:** *PEMT* rs4646343 genotype alters plasma choline PC-d3+6/PC-d9 enrichment ratios. Values are least-squared means ± standard errors for each group. Values are ratios.

Metabolic Outcome	WT	Variant	*p*-Value
**PC-d_3+6_/PC-d_9_**	0.32 ^±0.009^	0.30 ^±0.008^	0.05

**Table 11:** Comparison of PC-d3+6/PC-d9 enrichment ratios between *PEMT* rs7946 genotype by reproductive state. Values are least-squared means ± standard errors.

Metabolic Outcome and Group	WT	Variant	*p*-Value
**PC-d_3+6_/PC-d_9_ RS × Gene Interaction; *p* = 0.09**			
Lactating	0.37 ^±0.03^	0.29 ^±0.01^	0.03
Non-pregnant	0.32 ^±0.02^	0.33 ^±0.01^	>0.99
Pregnant	0.30 ^±0.04^	0.29 ^±0.01^	>0.99

**Table 12:** *PEMT* rs7946 genotype alters the metabolic flux of choline-derived methionine → PEMT-PC in plasma. Values are least-squared means ± standard errors for each group in µM PC/study period.

Metabolic Outcome	WT	Variant	*p*-Value
**Choline-Derived Methionine → PEMT-PC**	7942 ^±765^	9481 ^±312^	0.07

**Table 13:** *FMO3* rs2266782 genotype alters the metabolic flux of plasma choline metabolites. Values are least-squared means ± standard errors for each group. Betaine → methionine values are in µM methionine/study period, choline → betaine values are in µM betaine/study period and choline-derived methionine → PEMT-PC values are in µM PC/study period.

Metabolic Outcome	WT	Variant	*p*-Value
**Betaine → Methionine**	1.8 ^±0.05^	1.6 ^±0.05^	0.03
**Choline-Derived Methionine → PEMT-PC**	8609 ^±433^	9761 ^±384^	0.05

**Table 14:** Comparison of choline → CDP-PC turnover between *SLC44A1* rs7873987 genotypes by choline intake group. Values are least-squared means ± standard errors in µM methionine/study period.

Metabolic Outcome and Group	480 mg Choline/day	930 mg Choline/day	*p*-Value
**Betaine → Methionine Cho × Gene Interaction; *p* = 0.06**			
WT	1.60 ^±0.06^	1.70 ^±0.06^	0.9
Variant	1.51 ^±0.12^	1.95 ^±0.11^	0.03

**Table 15:** *SLC44A1* rs7873987 genotype alters the metabolic flux of betaine → DMG. Values are least-squared means ± standard errors in µM DMG/study period. *p*-values represent the highest order interaction and pairwise comparisons between genotypes within intake groups.

Metabolic Outcome and Group	480 mg Choline/day WT	480 mg Choline/day Variant	*p*-Value	930 mg Choline/day WT	930 mg Choline/day Variant	*p*-Value
**Betaine → DMG 3-Way Interaction; *p* = 0.04 **						
Lactating	6.2 ^±1.5^	6.7 ^±3.3^	>0.99	7.4 ^±1.5^	5.7 ^±3.3^	>0.99
Non-pregnant	9.2 ^±1.6^	5.6 ^±3.3^	>0.99	7.5 ^±1.5^	19.6 ^±3.3^	0.02
Pregnant	3.8 ^±1.5^	4.5 ^±2.3^	>0.99	5.6 ^± 1.4^	7.1 ^±3.3^	>0.99

**Table 16:** *SLC44A1* rs7873987 genotype alters the metabolic flux and partitioning of dietary choline. Values are least-squared means ± standard errors. Betaine → DMG values are in µM DMG/study period. *p*-values represent the highest order interaction and pairwise comparisons between genotypes within intake groups.

Metabolic Outcome and Group	480 mg Choline/day WT	930 mg Choline/day WT	*p*-Value	480 mg Choline/day Variant	930 mg Choline/day Variant	*p*-Value
**Betaine → DMG 3-Way Interaction; *p* = 0.04**						
Lactating	6.2 ^±1.5^	7.4 ^±1.5^	>0.99	6.7 ^±3.3^	5.7 ^±3.3^	>0.99
Non-pregnant	9.2 ^±1.6^	7.5 ^±1.5^	>0.99	5.6 ^±3.3^	19.6 ^±3.3^	0.05
Pregnant	3.8 ^±1.5^	5.6 ^±1.4^	>0.99	4.5 ^±2.3^	7.1 ^±3.3^	>0.99
**Choline-Derived Methionine → PEMT-PC 3-Way Interaction; *p* = 0.05**						
Lactating	8759 ^±741^	8197 ^±782^	>0.99	9648 ^±1658^	12417 ^±1658^	>0.99
Non-pregnant	9772 ^±829^	9331 ^±782^	>0.99	9257 ^±1658^	9172 ^±1658^	>0.99
Pregnant	9660 ^±782^	8900 ^±707^	>0.99	11635 ^±1172^	5780 ^±1658^	0.07
**PC-d_3+6_/PC-d_9_ 3-Way Interaction; *p* = 0.08**						
Lactating	0.27 ^±0.02^	0.34 ^±0.02^	0.05	0.21 ^±0.04^	0.39 ^± 0.04^	0.02
Non-pregnant	0.28 ^±0.02^	0.37 ^±0.02^	0.005	0.31 ^±0.04^	0.35 ^±0.04^	>0.99
Pregnant	0.25 ^±0.02^	0.33 ^±0.02^	0.006	0.26 ^±0.03^	0.29 ^±0.04^	>0.99

**Table 17:** Comparison of choline → CDP-PC turnover between *SLC44A1* rs3199966 genotypes by choline intake group. Values are least-squared means ± standard errors in µM methionine/study period.

Metabolic Outcome and Group	480 mg Choline/day	930 mg Choline/day	*p*-Value
**Betaine → Methionine Cho × Gene Interaction; *p* = 0.08**			
WT	1.60 ^±0.06^	1.70 ^±0.06^	>0.99
Variant	1.52 ^±0.10^	1.90 ^±0.11^	0.04

## Supplementary Materials

Supplementary materials can be found at www.mdpi.com/1422-0067/18/2/252/s1.

## Abbreviations

AI	Adequate intake
BHMT	Betaine homocysteine methyltransferase
CDP	Cytidine diphosphate
CHKA	Choline kinase-α
CHDH	Choline dehydrogenase
CI	Confidence interval
DMG	Dimethylglycine
FMO3	Flavin monooxygenase isoform 3
GC-MS	Gas chromatography-mass spectrometry
NTD	Neural tube defect
PE	Phosphatidylethanolamine
PEMT	Phosphatidylethanolamine *N*-methyltransferase
PC	Phosphatidylcholine
REV	SNP is identified on the reverse strand
RS	Reproductive state
SAH	*S*-adenosylhomocysteine
SAM	*S*-adenosylmethionine
SLC44A1	Solute carrier 44A1
MTHFR	Methylene tetrahydrofolate reductase
NAFLD	Non-alcoholic fatty liver disease
NP	Non-pregnant
WT	Wildtype

## References

[1] Caudill M.A., Miller J.W., III J.F.G. (2012). Folate, choline, vitamin B 12, and vitamin B 6. Stipanuk MH and Caudill MA. Biochemical, Physiological and Molecular Aspects of Human Nutrition.

[2] Li Z., Vance D.E. (2008). Phosphatidylcholine and choline homeostasis. J. Lipid Res..

[3] Delong C.J., Shen Y., Michael J., Cui Z. (1999). Molecular Distinction of Phosphatidylcholine Synthesis between the CDP-Choline Pathway and Pathway Molecular Distinction of Phosphatidylcholine Synthesis between the CDP-Choline Pathway and Phosphatidy. J. Biol. Chem..

[4] Institute of Medicine (US) Standing Committee on the Scientific Evaluation of Dietary Reference Intakes and its Panel on Folate, Other B Vitamins, and C (1998). Dietary Reference Intakes for Thiamin, Riboflavin, Niacin, Vitamin B_6_, Folate, Vitamin B_12_, Pantothenic Acid, Biotin, and Choline.

[5] Yan J., Wang W., Iii J.F.G., Malysheva O., Brenna J.T., Stabler S.P., Allen R.H., Caudill M.A. (2011). MTHFR C677T genotype influences the isotopic enrichment of one-carbon metabolites in folate-compromised men consuming. Am. J. Clin. Nutr..

[6] Yan J., Jiang X., West A.A., Perry C.A., Malysheva O.V., Devapatla S., Pressman E., Vermeylen F., Stabler S.P., Allen R.H. (2012). Maternal choline intake modulates maternal and fetal biomarkers of choline metabolism in humans. Am. J. Clin. Nutr..

[7] Fischer L.M., DaCosta K.A., Kwock L., Stewart P.W., Lu T.S., Stabler S.P., Allen R.H., Zeisel S.H. (2007). Sex and menopausal status influence human dietary requirements for the nutrient choline. Am. J. Clin. Nutr..

[8] Da Costa K.-A., Kozyreva O.G., Song J., Galanko J.A., Fischer L.M., Zeisel S.H. (2006). Common genetic polymorphisms affect the human requirement for the nutrient choline. FASEB J..

[9] Ganz A.B., Shields K., Fomin V.G., Lopez Y.S., Mohan S., Lovesky J., Chuang J.C., Ganti A., Carrier B., Yan J. (2016). Genetic impairments in folate enzymes increase dependence on dietary choline for phosphatidylcholine production at the expense of betaine synthesis. FASEB J..

[10] Da Costa K.A., Corbin K.D., Niculescu M.D., Galanko J.A., Zeisel S.H. (2014). Identification of new genetic polymorphisms that alter the dietary requirement for choline and vary in their distribution across ethnic and racial groups. FASEB J..

[11] Jeff J.M., Armstrong L.L., Ritchie M.D., Denny J.C., Kho A.N., Basford M.A., Wolf W.A., Pacheco J.A., Li R., Chisholm R.L. (2014). Admixture mapping and subsequent fine-mapping suggests a biologically relevant and novel association on chromosome 11 for type 2 diabetes in African Americans. PLoS ONE.

[12] Schläwicke Engström K., Nermell B., Concha G., Strömberg U., Vahter M., Broberg K. (2009). Arsenic metabolism is influenced by polymorphisms in genes involved in one-carbon metabolism and reduction reactions. Mutat. Res..

[13] Xu X., Gammon M.D., Zeisel S.H., Lee Y.L., Wetmur J.G., Teitelbaum S.L., Bradshaw P.T., Neugut A.I., Santella R.M., Chen J. (2008). Choline metabolism and risk of breast cancer in a population-based study. FASEB J..

[14] Johnson A.R., Lao S., Wang T., Galanko J.A., Zeisel S.H. (2012). Choline dehydrogenase polymorphism rs12676 is a functional variation and is associated with changes in human sperm cell function. PLoS ONE.

[15] Mostowska A., Hozyasz K.K., Wojcicki P., Dziegelewska M., Jagodzinski P.P. (2010). Associations of folate and choline metabolism gene polymorphisms with orofacial clefts. J. Med. Genet..

[16] Xu X., Gammon M.D., Wetmur J.G., Bradshaw P.T., Susan L., Neugut A.I., Santella R.M., Chen J. (2009). NIH B-vitamin Intake, One-carbon Metabolism and Survival among a Population-based Study of Women with Breast Cancer. Biomarkers.

[17] Shaw G.M., Lu W., Zhu H., Yang W., Briggs F.B.S., Carmichael S.L., Barcellos L.F., Lammer E.J., Finnell R.H. (2009). 118 SNPs of folate-related genes and risks of spina bifida and conotruncal heart defects. BMC Med. Genet..

[18] Boyles A.L., Billups A.V., Deak K.L., Siegel D.G., Mehltretter L., Slifer S.H., Bassuk A.G., Kessler J.A., Reed M.C., Nijhout H.F. (2006). Neural tube defects and folate pathway genes: Family-based association tests of gene-gene and gene-environment interactions. Environ. Health Perspect..

[19] Sharma N.K., Langberg K.A., Mondal A.K., Das S.K. (2013). Phospholipid biosynthesis genes and susceptibility to obesity: Analysis of expression and polymorphisms. PLoS ONE.

[20] Song J., da Costa K.A., Fischer L.M., Kohlmeier M., Kwock L., Wang S., Zeisel S.H. (2005). Polymorphism of the *PEMT* gene and susceptibility to nonalcoholic fatty liver disease (NAFLD). FASEB J..

[21] Zeisel S.H. (2006). People with fatty liver are more likely to have the *PEMT* rs7946 SNP, yet populations with the mutant allele do not have fatty liver. FASEB J..

[22] Phillips I.R., Shephard E.A. (1993). Primary Trimethylaminuria.

[23] Gibellini F., Smith T.K. (2010). The Kennedy pathway-de novo synthesis of phosphatidylethanolamine and phosphatidylcholine. IUBMB Life.

[24] Trousil S., Lee P., Pinato D.J., Ellis J., Dina R., Aboagye E.O., Keun H.C., Sharma R. (2014). Alterations of choline phospholipid metabolism in endometrial cancer are caused by choline kinase α overexpression and a hyperactivated deacylation pathway. Cancer Res..

[25] Ramírez de Molina A., Gallego-Ortega D., Sarmentero-Estrada J., Lagares D., Gómez del Pulgar T., Bandrés E., García-Foncillas J., Lacal J.C. (2008). Choline kinase as a link connecting phospholipid metabolism and cell cycle regulation: Implications in cancer therapy. Int. J. Biochem. Cell Biol..

[26] Gréchez-Cassiau A., Feillet C., Guérin S., Delaunay F. (2015). The hepatic circadian clock regulates the choline kinase **α** gene through the BMAL1-REV-ERB α axis. Chronobiol. Int..

[27] Jacobs R.L., Zhao Y., Koonen D.P.Y., Sletten T., Su B., Lingrell S., Cao G., Peake D.A., Kuo M.-S., Proctor S.D. (2010). Impaired de novo choline synthesis explains why phosphatidylethanolamine *N*-methyltransferase-deficient mice are protected from diet-induced obesity. J. Biol. Chem..

[28] Zeisel S.H. (2007). Gene response elements, genetic polymorphisms and epigenetics influence the human dietary requirement for choline. IUBMB Life.

[29] Wang Z., Dahiya S., Provencher H., Muir B., Carney E., Coser K., Shioda T., Ma X.-J., Sgroi D.C. (2007). The Prognostic Biomarkers HOXB13, IL17BR, and CHDH Are Regulated by Estrogen in Breast Cancer. Clin. Cancer Res..

[30] Li F., Feng Q., Lee C., Wang S., Pelleymounter L.L., Moon I., Eckloff B.W., Wieben E.D., Schaid D.J., Yee V. (2008). Human betaine-homocysteine methyltransferase (BHMT) and BHMT2: Common gene sequence variation and functional characterization. Mol. Genet. Metab..

[31] Morin I., Platt R., Weisberg I., Sabbaghian N., Wu Q., Garrow T.A., Rozen R. (2003). Common variant in betaine-homocysteine methyltransferase (BHMT) and risk for spina bifida. Am. J. Med. Genet. A.

[32] Ridgway N.D., Vance D.E. (1988). Kinetic mechanism of phosphatidylethanolamine *N*-methyltransferase. J. Biol. Chem..

[33] Resseguie M.E., da Costa K.-A., Galanko J.A., Patel M., Davis I.J., Zeisel S.H. (2011). Aberrant estrogen regulation of PEMT results in choline deficiency-associated liver dysfunction. J. Biol. Chem..

[34] Pynn C.J., Henderson N.G., Clark H., Koster G., Bernhard W., Postle A.D. (2011). Specificity and rate of human and mouse liver and plasma phosphatidylcholine synthesis analyzed in vivo. J. Lipid Res..

[35] Wallace T.C., Fulgoni V.L. (2016). Assessment of Total Choline Intakes in the United States. J. Am. Coll. Nutr..

[36] Craciun S., Balskus E.P. (2012). Microbial conversion of choline to trimethylamine requires a glycyl radical enzyme. Proc. Natl. Acad. Sci. USA.

[37] Koukouritaki S.B., Poch M.T., Cabacungan E.T., McCarver D.G., Hines R.N. (2005). Discovery of novel flavin-containing monooxygenase 3 (FMO3) single nucleotide polymorphisms and functional analysis of upstream haplotype variants. Mol. Pharmacol..

[38] Miao J., Ling A.V., Manthena P.V., Gearing M.E., Graham M.J., Crooke R.M., Croce K.J., Esquejo R.M., Clish C.B., Torrecilla E. (2015). Flavin-containing monooxygenase 3 as a potential player in diabetes-associated atherosclerosis. Nat. Commun..

[39] Bennett B.J., Vallim T.Q.A., Wang Z., Shih D.M., Meng Y., Gregory J., Allayee H., Lee R., Graham M., Crooke R. (2012). Trimethylamine-N-Oxide, a Metabolite Associated with Atherosclerosis, Exhibits Complex Genetic and Dietary Regulation. Changes.

[40] Tang W.H.W., Wang Z., Levison B.S., Koeth R.A., Britt E.B., Fu X., Wu Y., Hazen S.L. (2013). Intestinal microbial metabolism of phosphatidylcholine and cardiovascular risk. N. Engl. J. Med..

[41] Koeth R.A., Wang Z., Levison B.S., Buffa J.A., Org E., Sheehy B.T., Britt E.B., Fu X., Wu Y., Li L. (2013). Intestinal microbiota metabolism of l-carnitine, a nutrient in red meat, promotes atherosclerosis. Nat. Med..

[42] Warrier M., Shih D.M., Burrows A.C., Ferguson D., Gromovsky A.D., Brown A.L., Marshall S., McDaniel A., Schugar R.C., Wang Z. (2015). The TMAO-Generating Enzyme Flavin Monooxygenase 3 Is a Central Regulator of Cholesterol Balance. Cell Rep..

[43] Cho C.E., Taesuwan S., Malysheva O.V., Bender E., Yan J., Caudill M.A. (2016). Choline and one-carbon metabolite response to egg, beef and fish among healthy young men: A short-term randomized clinical study. Clin. Nutr. Exp..

[44] PB|CTL1–5 (Plasma Membrane). http://www.reactome.org/PathwayBrowser/#/R-HSA-425366&SEL=R-HSA-444452.

[45] SLC44A1; Choline Transporter-Like Protein 1. https://www.nextprot.org/entry/NX_Q8WWI5/.

[46] Traiffort E., O’Regan S., Ruat M. (2013). The choline transporter-like family SLC44: Properties and roles in human diseases. Mol. Asp. Med..

[47] Michel V., Singh R.K., Bakovic M. (2011). The impact of choline availability on muscle lipid metabolism. Food Funct..

[48] R Core Team (2016). R: A Language and Environment for Statistical Computing, 2014.

