# Supplementary material for: Genetic Variation in Choline-Metabolizing Enzymes Alters Choline Metabolism in Young Women Consuming Choline Intakes Meeting Current Recommendations
Source: Int J Mol Sci. 2017 Jan 26;18(2):252. doi: 10.3390/ijms18020252 (PMC5343788; doi:10.3390/ijms18020252)
Supplement: Supplementary file 1 [file ijms-18-00252-s001.pdf]

Supplementary Materials: Genetic Variation in Choline-Metabolizing Enzymes Alters Choline Metabolism in Young Women Consuming Choline Intakes Meeting Current Recommendations

Ariel B. Ganz, Vanessa V. Cohen, Camille C. Swersky, Julie Stover, Gerardo A. Vitiello, Jessica Lovesky, Jasmine C. Chuang, Kelsey Shields, Vladislav G. Fomin, Yusnier S. Lopez Sanjay Mohan, Anita Ganti, Bradley Carrier, Olga V. Malysheva and Marie A. Caudill

Table S1. Effect of SNP genotype and interaction (*p*-values) on plasma choline metabolite partitioning and flux.

SNP and Metabolic Outcome	Gene	G × RS	G × Cho	Three-Way
***CHKA* rs10791957**				
Betaine-d_9/PC-d_9	0.6	-	-	-
PC-d_{3+6}/PC-d_9	0.09	-	-	-
Choline → Betaine	0.8	-	-	-
Choline → CDP-PC	0.3	-	-	-
Betaine → DMG	0.5	-	-	-
Betaine → Methionine	0.6	-	-	-
Methionine → PEMT-PC	0.0005	-	-	-
***CHDH* rs9001**				
Betaine-d_9/PC-d_9	0.2	-	0.07	-
PC-d_{3+6}/PC-d_9	0.1	-	-	-
Choline → Betaine	0.6	-	-	-
Choline → CDP-PC	0.7	0.04		
Betaine → DMG	0.2	-	-	-
Betaine → Methionine	0.9	-	-	-
Methionine → PEMT-PC	0.9	-	-	-
***CHDH* rs12676**				
Betaine-d_9/PC-d_9	0.2	-	-	-
PC-d_{3+6}/PC-d_9	0.055	-	-	-
Choline → Betaine	0.3	-	-	-
Choline → CDP-PC	0.2	0.08	0.9	0.05
Betaine → DMG	0.03	0.09	-	-
Betaine → Methionine	0.2	-	0.1	-
Methionine → PEMT-PC	0.2	0.02	0.8	0.05
***BHMT* rs3733890**				
Betaine-d_9/PC-d_9	0.066	-	-	-
PC-d_{3+6}/PC-d_9	0.3	-	-	-
Choline → Betaine	0.03	-	-	-
Choline → CDP-PC	0.03	-	-	-
Betaine → DMG	0.1	-	-	-
Betaine → Methionine	0.95	-	-	-
Methionine → PEMT-PC	0.15	-	-	-

Table S1. *Cont.*

SNP and Metabolic Outcome	Gene	G × RS	G × Cho	Three-Way
***PEMT* rs4646343**				
Betaine-d_9/PC-d_9	0.6	-	-	-
PC-d_{3+6}/PC-d_9	0.05	-	-	-
Choline → Betaine	0.9	-	-	-
Choline → CDP-PC	0.9	-	-	-
Betaine → DMG	0.6	-	-	-
Betaine → Methionine	0.97	0.08	-	-
Methionine → PEMT-PC	0.6	0.4	0.2	0.04
***PEMT* rs7946**				
Betaine-d_9/PC-d_9	0.1	-	-	-
PC-d_{3+6}/PC-d_9	0.1	0.098	-	-
Choline → Betaine	0.96	-	-	-
Choline → CDP-PC	0.5	-	-	-
Betaine → DMG	0.3	-	-	-
Betaine → Methionine	0.3	-	-	-
Methionine → PEMT-PC	0.07	-	-	-
***FMO3* rs2266782**				
Betaine-d_9/PC-d_9	0.1	-	-	-
PC-d_{3+6}/PC-d_9	0.6	-	-	-
Choline → Betaine	0.9	-	-	-
Choline → CDP-PC	0.9	-	-	-
Betaine → DMG	0.7	-	-	-
Betaine → Methionine	0.03	-	-	-
Methionine → PEMT-PC	0.05	-	-	-
***SLC44A1* rs7873937**		-		
Betaine-d_9/PC-d_9	0.5	-	-	-
PC-d_{3+6}/PC-d_9	0.8	0.96	0.8	0.08
Choline → Betaine	0.3	-	-	-
Choline → CDP-PC	0.7	-	-	-
Betaine → DMG	0.3	0.4	0.10	0.04
Betaine → Methionine	0.6	-	0.06	-
Methionine → PEMT-PC	0.3	0.2	0.5	0.05
***SLC44A1* rs3199966**		-	-	-
Betaine-d_9/PC-d_9	0.9	-	-	-
PC-d_{3+6}/PC-d_9	0.4	-	-	-
Choline → Betaine	0.2	-	-	-
Choline → CDP-PC	0.2	-	-	-
Betaine → DMG	0.8	0.5	0.5	0.106
Betaine → Methionine	0.5	-	0.08	-
Methionine → PEMT-PC	0.7	-	-	-
